# The key role of nanoparticle concentration gradient in aerosol initial growth

**DOI:** 10.1038/s41467-026-70082-2

**Published:** 2026-03-02

**Authors:** Runlong Cai, Xiaoxiao Li, Yuyang Li, Sara Blichner, Dominik Stolzenburg, Qiaozhi Zha, Jing Cai, Wei Nie, Chao Yan, Dan Dan Huang, Zhe Wang, Jin Wu, Rujing Yin, Nina Sarnela, Wei Huang, Santeri Tuovinen, Sebastian Holm, Lauri Ahonen, Lei Yao, Aijun Ding, Federico Bianchi, Yongchun Liu, Paul M. Winkler, Tuukka Petäjä, Jianmin Chen, Veli-Matti Kerminen, Lin Wang, Douglas Worsnop, Jingkun Jiang, Markku Kulmala, Juha Kangasluoma

**Affiliations:** 1 Shanghai Key Laboratory of Air Quality and Environmental Health, Department of Environmental Science & Engineering, Fudan University, Shanghai, China; 2 Institute for Atmospheric and Earth System Research / Physics, Faculty of Science, University of Helsinki, Helsinki, Finland; 3 IRDR ICoE on Risk Interconnectivity and Governance on Weather/Climate Extremes Impact and Public Health, Fudan University, Shanghai, China; 4 School of Resource and Environmental Sciences, Wuhan University, Wuhan, China; 5 State Key Laboratory of Regional Environment and Sustainability, School of Environment, Tsinghua University, Beijing, China; 6 Bolin Centre for Climate Research, Stockholm University, Stockholm, Sweden; 7 Department of Environmental Science, Stockholm University, Stockholm, Sweden; 8 Institute of Materials Chemistry, TU Wien, Vienna, Austria; 9 Joint International Research Laboratory of Atmospheric and Earth System Research, School of Atmospheric Sciences, Nanjing University, Nanjing, China; 10 School of Atmospheric Physics, Nanjing University of Information Science and Technology, Nanjing, China; 11 State Environmental Protection Key Laboratory of Formation and Prevention of Urban Air Pollution Complex, Shanghai Academy of Environmental Sciences, Shanghai, China; 12 Division of Environment and Sustainability, The Hong Kong University of Science and Technology, Hong Kong, China; 13 Key Laboratory of Industrial Ecology and Environmental Engineering (Ministry of Education), School of Environmental Science and Technology, Dalian University of Technology, Dalian, China; 14 PSI Center for Energy and Environmental Sciences, Villigen PSI, Switzerland; 15 Aerosol and Haze Laboratory, Beijing Advanced Innovation Center for Soft Matter Science and Engineering, Beijing University of Chemical Technology, Beijing, China; 16 Faculty of Physics, University of Vienna, Vienna, Austria; 17 Aerodyne Research Inc., 45 Manning Road, Billerica, MA USA

**Keywords:** Atmospheric chemistry, Atmospheric chemistry

## Abstract

New particle formation has been estimated to produce more than half of the global cloud condensation nuclei and profoundly impacts clouds, climate, and air quality. The initial growth from the cluster size ( ~ 1 nm) to a few nanometers, for which the underlying mechanisms can be very different from the subsequent growth, is the most critical stage for new particles to become climate-relevant. However, initial growth mechanisms evidenced by controlled laboratory experiments can rarely explain observations from the real atmosphere. Here we show that a large nanoparticle concentration gradient in the size space can drive unexpected rapid initial growth based on measurements across the globe. It accelerates the condensation of globally abundant oxygenated organic molecules onto a population of new particles compared to a single particle, and substantially increases the fraction of new particles that survive to climate- and air-quality-relevant sizes. Our findings provide insights into explaining the puzzle of the frequent new particle formation events in polluted urban environments and indicate an even more important role of new particle formation in climate predictions.

## Abstract (web summary)

The growth of the smallest aerosol particles is critical to their impacts on climate and air quality. Here, the concentration gradient of nanoparticles is shown to be an important microphysical mechanism for their rapid initial growth.

## Introduction

Atmospheric aerosols mask a significant portion of radiative forcing by greenhouse gases since the Industrial Revolution^1^. New particles formed by nucleation and growth contribute more than 50% of the global cloud condensation nuclei (CCN) and they have persistently been a large source of uncertainties in estimating the effective radiative forcing from aerosols^2,3^. With a significantly improved understanding of atmospheric nucleation^4,5^, how freshly nucleated particles grow and become relevant (e.g., 50 or 100 nm in diameter) for climate and air quality^6,7^ remains uncertain. The initial growth, defined here as the growth from the size of ~ 1 nm to 3 nm, is a critical stage that determines the fraction of freshly nucleated particles surviving to large sizes, as particles in this “death zone” are most susceptible to coagulation scavenging^8^. Due to small particle mass, strong Kelvin effect, and minimal particle-phase diffusion of sub-3 nm particles, the initial growth mechanism remains ambiguous as it can be fundamentally different from that derived for larger particles (e.g., 10 nm)^9^.

Previous well-controlled laboratory experiments have shown that the initial growth could be explained by vapor condensation and the growth rate responds linearly to the concentration of condensable vapors^10,11^. However, it is a puzzling mystery that the good consistency between condensable vapors and initial growth rate in lab experiments does not apply to the real atmosphere^12^, in which the measured initial growth rate exhibits a rather weak dependence on the condensable vapor concentration (Supplementary Information Fig. 1). Besides, the limited concentration of condensable vapors often does not explain the observed rapid initial growth. Sulfuric acid, ubiquitous in various atmospheric environments with a typical concentration of 10^4^–10^7^ molecules cm^-3^,^13^ can explain events with slow initial growth ( ~ 1 nm h^-1^). However, it can rarely explain rapid initial growth ( ~ 3 nm h^-1^) even after considering the growth enhancement of intermolecular forces^10^. The extremely low volatility (saturation concentration < 10^−4.5^ μg m^-3^, corresponding to ~10^5^ molecules cm^-3^) fraction of highly-oxygenated organic molecules (HOMs)^14^ has been proposed to be a major contributor to the initial growth as they can overcome the significant Kelvin effect^11,15^. However, the concentration of extremely low-volatility HOMs is usually low, e.g., ~10^7^ molecules cm^-3^ during NPF events in urban Beijing^16^. With the limited availability of condensable vapors, slow initial growth of sub-3 nm particles was expected^17,18^, which could not explain the observed rapid initial growth rate^19^ (see methods for discussions in detail) and the large fraction of freshly nucleated particles surviving to larger size^20^.

Here, we propose that this mystery of initial growth can be addressed by an underrepresented nanoparticle concentration gradient (NCG) in the size space. For NPF in the real atmosphere, a population of freshly nucleated particles grows more rapidly than a single particle as a result of a strong gradient in their concentrations as a function of particle size, which significantly accelerates vapor condensation, even below their corresponding Kelvin diameter of nanoparticles. We further show that accounting for the contribution of NCG to nanoparticle initial growth provides a consistent prediction of the measured survival probability of new atmospheric particles and hence the puzzling frequent NPF events in polluted urban environments. Analysis of data from seven sites across the globe shows that NCG doubles the initial growth rate of oxygenated organic molecules (OOMs) and substantially reduces new particle loss by a factor of ~2 to several orders of magnitude. This significantly increases the estimated contribution of NPF to regional CCN budgets, especially in urban environments and downwind areas, and even possibly in the remote atmosphere.

## Results and discussion

### Rapid initial growth of new atmospheric particles

**Fig. 1:**
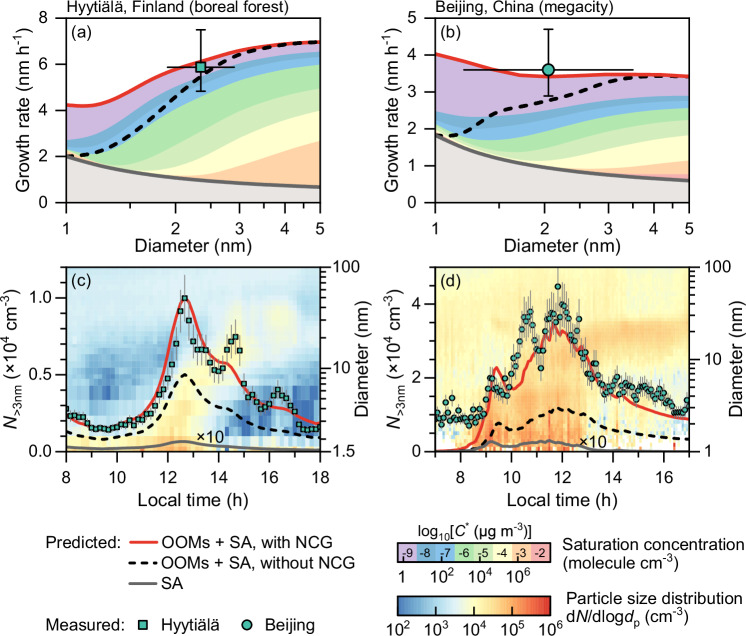
The initial growth rate and nanoparticle concentration during atmospheric NPF events at Hyytiälä and Beijing. **a**, **b** the measured and theoretically predicted growth rates of freshly nucleated particles during new particle formation (NPF) events with and without the nanoparticle concentration gradient (NCG). The marker indicates the measured growth rate. The vertical uncertainty bar indicates the uncertainty in determining the growth rate for the measured temporal evolution of particle size distributions. The horizontal variation bar indicates the size range of particles used to determine the measured growth rate. The shaded area indicates contributions from sulfuric acid (SA) (gray) and oxygenated organic molecules (OOMs) with different volatilities. **c**, **d** the measured and theoretically predicted concentrations of particles larger than 3 nm (*N*_>3nm_). The uncertainty bar indicates an estimated 15% uncertainty in the measured concentration. The predicted *N*_>3nm_ was obtained using the new particle formation rate and the survival probability corresponding to the predicted growth rates. Note that the curves for only sulfuric-acid-driven growth in (**c**) and (**d**) have been multiplied by 10 for visualization.

We observed rapid initial growth of new atmospheric particles (see Methods) at a boreal forest site^17^ (Hyytiälä, Finland, March – May 2020) and a megacity site^21^ (Beijing, China, January 2018 – March 2019) and investigated the growth mechanism by contrasting the predicted growth rates and particle number concentrations from condensable vapors to measured values. Figure 1a and b show that the measured initial growth rates retrieved were much higher than what could be explained by sulfuric acid, despite that we took sulfuric acid as a non-volatile vapor and accounted for the collision enhancement due to van der Waals forces^10^. We further found that predictions accounting for OOM condensation could explain the measured growth rate, indicating that OOMs governed the rapid initial growth. The governing role of OOMs in initial growth contrasts with the previous understanding of sulfuric acid-governed initial growth of particles formed by acid-base clustering in polluted megacities^22–24^. Figure 1a, b also show that the NCG (elucidated in the following section) accelerates the initial growth as an aerosol population compared to a single particle, making a major difference in the initial growth of the smallest particles at both sites.

The rapid initial growth exerts a strong influence on the concentration of new particles. Figure 1c, d show the measured concentrations of particles larger than 3 nm during the measured NPF events and the predictions based on the surviving new particles. A discrepancy of 2–4 orders of magnitude between measurements and predictions manifests that sulfuric acid (4.0×10^6^ molecules cm^-3^ for this event) alone cannot grow freshly nucleated particles to large sizes before they are scavenged, even in sulfur-rich environments such as urban Beijing. Accounting for NCG's contribution to OOM condensation, we observed good agreement between measured and predicted particle concentrations. For the NPF event in Fig. 1d, the NCG significantly improves the number of new particles surviving from scavenging losses by contributing to the initial growth rate (discussed in detail below).

### Initial growth contributed by NCG

**Fig. 2:**
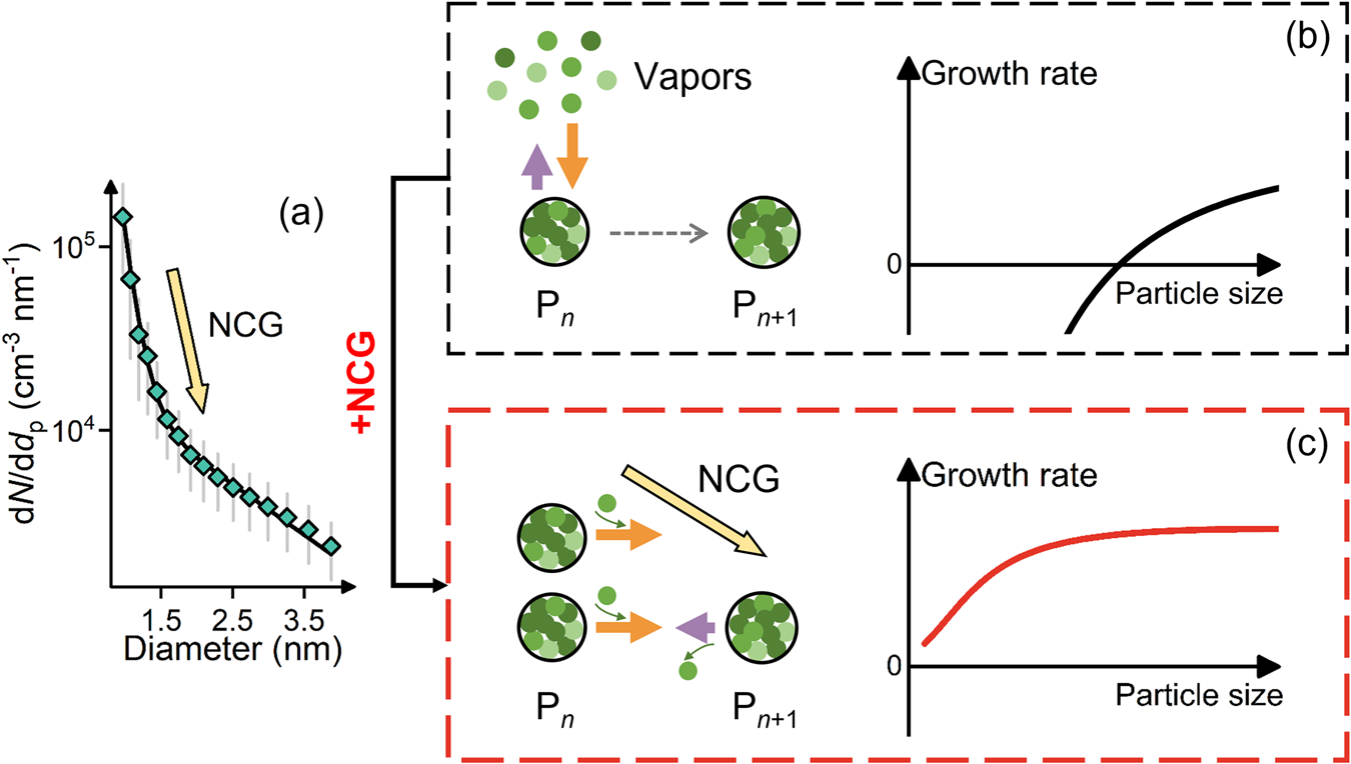
Illustrative schematic for the initial growth of a single particle and a population of particles. **a** number size distribution of new atmospheric particles averaged between 11-13 am (local time) during NPF events measured in Beijing. The variation bar indicates the relative variation in size distribution after normalizing particle concentration. **b** schematics illustrating the initial growth in a single-particle perspective. P_*n*_ represents particles containing *n* molecules. Green circles represent different condensable molecules. The solid arrows indicate association and dissociation processes. **c** schematics illustrating the growth flux of a population of particles. The yellow arrow indicates the nanoparticle concentration gradient (NCG) that enhances the initial growth.

We illustrate particle growth by vapor condensation in Fig. 2 and show that a large NCG of freshly nucleated particles in the size space (Fig. 2a) drives rapid initial growth. When evaluating the contribution of vapor condensation to particle growth from measurements or in large-scale models^5,11^, the growth rate is usually determined using the balance between the surrounding vapors and the molecules in a single particle, or equivalently, a collection of strictly monodisperse aerosol particles^25,26^. In such a single-particle perspective, a particle tends to evolve towards larger sizes when the vapor concentration is sufficiently high to overcome the Kelvin effect, i.e., the rate of vapor association exceeds dissociation (Fig. 2b).

However, a population of new particles tends to disperse into different sizes, and their growth is determined by the net rate of particles passing through a given diameter. NCG, the gradient in nanoparticle concentration as a function of size, results from significant vapor dissociation and coagulation scavenging during initial growth. Previous theoretical analysis indicates that NCG may contribute substantially to particle growth^27–31^, and a similar mechanism has been proposed for drizzle formation^32^. However, the importance of NCG in atmospheric nanoparticle growth has been overlooked due to a lack of measurement evidence. Here our observations show that there is usually a large negative NCG during atmospheric NPF (Fig. 2a). The lower concentration of larger particles causes lower dissociation flux compared to that in a single-particle perspective, resulting in more effective condensation of volatile vapors^33^ (Fig. 2c). The weakened Kelvin effect of larger particles further increases the effectiveness of vapor condensation and enables growth below the Kelvin diameter (Supplementary Information Fig. 2).

The difference between single-particle growth and population growth of freshly nucleated particles is further illustrated in Supplementary Information Fig. 3. From a kinetic perspective, the NCG accelerates the initial growth rate of a collection of particles compared to single-particle growth. A negative OOM-driven growth rate of a single particle is expected at the smallest sizes because the Kelvin effect elevates the equilibrium concentration. Consequently, every single particle is more likely to evaporate than grow by OOMs. However, with a large negative NCG, there are more particles passing through a given diameter by vapor association than particles evaporating back by vapor dissociation, corresponding to an extra NCG term for the initial growth (see Eq. 5). This NCG term compensates for the negative single-particle growth rate and results in net population growth by OOM condensation below the Kelvin diameter.

### Initial growth and survival of new atmospheric particles

**Fig. 3:**
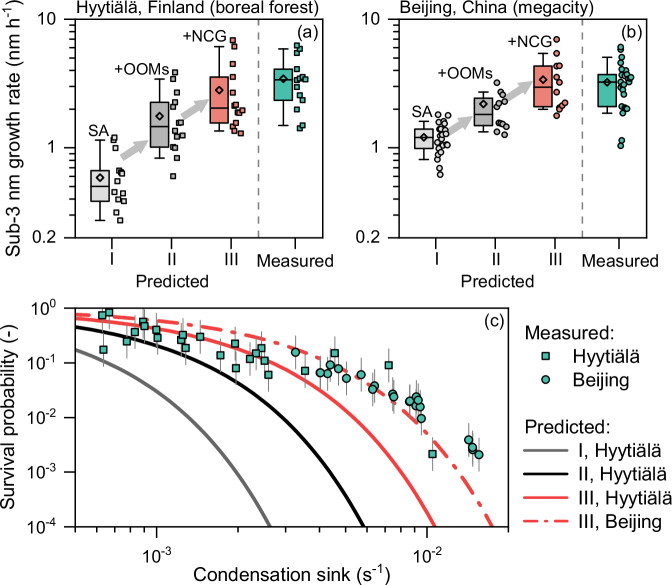
Statistical results of the growth rate and survival probability of sub-3 nm particles. **a**, **b** the measured and theoretically predicted growth rates during NPF events. The diamond marker, horizontal line, box, and whisker of the box plot indicate mean, median, 25th-75th percentiles, and 10th-90th percentiles of the data. I, II, and III represent growth by sulfuric acid, growth by sulfuric acid (SA) and oxygenated organic molecules (OOMs) without the nanoparticle concentration gradient (NCG), and growth by sulfuric acid and OOMs with the contribution of NCG, respectively. The contribution of NCG was analyzed for 26 events. The vertical dashed line is used to separate the measured and predicted growth rates. There are fewer predicted growth rates than measured growth rates in (**b**) because of the absence of the measured vapor concentrations, yet we find that the statistical results are not sensitive to the selection of NPF days. **c** The measured and theoretically predicted survival probabilities of particles from 1.5 to 3 nm. The uncertainty bar indicates a + 100% / −50% uncertainty in the measured survival probability. The theoretical survival probabilities are predicted using the mean values of predicted growth rates in (**a**) and (**b**). The predicted lines for I and II in Beijing are shown in Supplementary Information Fig. 5 for better visualization.

Consistent with the observed rapid initial growth, the NCG drives more effective condensation of OOMs than previously expected from the perspective of a single particle^11,26^. Figure 1a, b shows that the OOM condensation by the NCG was most effective for the smallest particles and it explained a considerable fraction of the initial growth rate for Hyytiälä and Beijing. Figure 3a, b further show that these findings from case studies are supported by statistical analysis of 47 strong NPF events (Supplementary Information Table 1); though the predicted growth rate at Hyytiälä is, on average, lower than measurements, plausibly due to extra undetected vapors^34^. The measured growth rates in a few NPF events coincide with the sulfuric acid concentration, and previous studies^24,35^ have reported moderate initial growth rates ( ~ 1-2 nm h^-1^) that could be explained using sulfuric acid. However, the high initial growth rate observed in a majority of the NPF events, sometimes exceeding 5 nm h^-1^, could not be solely explained by the measured sulfuric acid with such a limited concentration ( < 1×10^7^ molecules cm^-3^). With the NCG-enhanced OOM condensation, the predicted growth rate is in line with the measurements. The difference between the single-particle growth and measurements could be hypothetically attributed to inefficient measurement of OOMs; however, here we show that the initial sub-3 nm growth driven by the NCG fills this gap well without introducing a large amount of extra undetected condensable vapors^15,34^ (see Methods for discussions in detail). Besides, using the NCG derived from the measured particle size distribution rather than condensable vapors, we also find a good consistency between the predicted and measured initial growth rate (Supplementary Information Fig. 4). That is, the measured NCG corresponds to faster initial growth than previously expected, which can explain the missing growth.

The OOM condensation enhanced by the NCG can elevate the survival probability of new atmospheric particles dramatically. Figure 3c shows that the survival probability of freshly nucleated particles from 1.5 to 3 nm is a strong function of their scavenging losses characterized by the condensation sink. Interestingly, yet consistent with our prediction, the measured survival probabilities at Hyytiälä and Beijing follow a similar decreasing trend as the condensation sink increases. The initial growth predicted using single-particle growth (case II) may explain particle survival at a low condensation sink ( < 1 × 10^-3 ^s^-1^), however, it shows a large deviation from the measurements at moderate and high condensation sinks ( > 5 × 10^-3 ^s^-1^), where rapid initial growth is needed for sub-3 nm to survive. Accounting for the contribution of NCG to the initial growth provides good agreement between predicted and measured survival probabilities (case III). An enhancement in the initial growth rate by a multiplicative factor of 2 corresponds to a decrease in the number of scavenged particles by 2^n^ (n > 1, positively correlated with the condensation sink). With such a highly non-linear relationship, the NCG can elevate the survival probability considerably even in the relatively clean boreal forest environment at Hyytiälä (e.g., a factor of 4 at a condensation sink of 2 × 10^-3 ^s^-1^), and this elevation can be several orders of magnitude for NPF with a high condensation sink in polluted megacities (Fig. 3c and Supplementary Information Fig. 5).

Growth has conventionally been taken as a separate process that occurs after nucleation. Such a simplification of the growth rate from a single-particle perspective can be reasonable for controlled systems involving a few key nucleating species. However, our above analysis indicates that it can be impractical to separate the nucleation and growth stages unambiguously for NPF in a real atmosphere, as NCG contributes to particle initial growth in a manner similar to nucleation^27,33^. Although clusters containing a few molecules can already be stable against the evaporation of nucleating vapors, they still need this decisive contribution from NCG to grow rapidly beyond the most susceptible sub-3 nm size range to coagulation scavenging.

### Atmospheric implications

Extensive efforts have been spent on unveiling the molecular-level mechanism of atmospheric nucleation from various vapors^4,36^, which provide foundations for mapping the important roles of NPF-produced aerosols in Earth system models^5^. The growth of freshly nucleated particles against the scavenging of background aerosol, however, can be arguably more important for the influences of NPF than nucleation^6^, with the initial growth from the cluster size to a few nanometers being the most critical. Our findings show that the OOM condensation enhanced by the NCG can drive rapid initial growth and thus governs the number of NPF-produced particles. Without the enhanced growth rate, a large fraction (e.g., on average 57% for Hyytiälä and 95% for Beijing) of freshly nucleated particles would be scavenged before they exert significant impacts on climate and air quality.

**Fig. 4:**
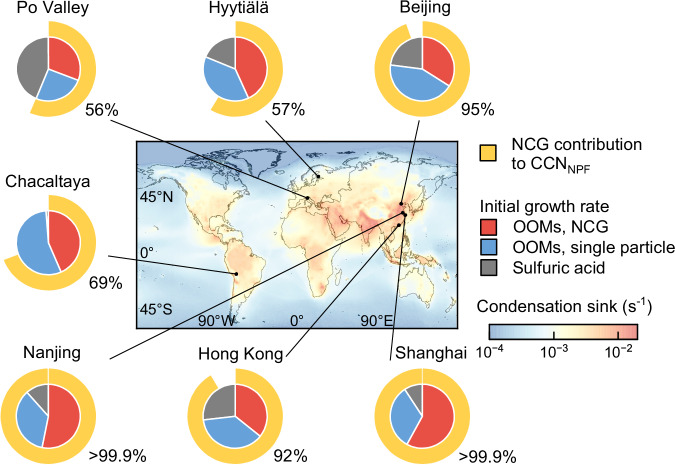
of the nanoparticle concentration gradient to the initial growth and survival of new particles at sites across the globe. Importance: The pie chart shows the average contribution determined using measured vapor concentration and condensation sink at every site. The inner ring of the pie chart shows the contributions of different mechanisms to sub-3 nm particle growth rate using the measured oxygenated organic molecules (OOMs) and sulfuric acid at seven sites^17,21,47–49^. The outer ring and label show the fraction of NPF-produced cloud condensation nuclei (CCN_NPF_, evaluated by the number concentration of 100-nm particles) that would be scavenged without the nanoparticle concentration gradient (NCG), e.g., 95% indicates that the NCG increases the survival probability by a factor of 20. The global map shows the spatial distribution of scavenging aerosols characterized by the condensation sink simulated using NorESM2^75^.

To assess the underrepresented importance of NPF associated with the rapid particle growth by NCG, we evaluate the growth and survival of freshly nucleated particles at seven sites across the globe. Figure 4 shows that OOMs govern the initial growth at all these sites including sulfur-rich urban sites where most new particles are formed by sulfuric acid-involved nucleation^22,37^. The NCG increases the initial growth rate by 45-138%. We find the contribution of the NCG to the initial growth rate is mainly determined by the concentration ratio of condensable OOMs to non-volatile vapors (sulfuric acid and ultra-low volatility vapors, see Supplementary Information Fig. 6), and it on average doubles the OOMs-driven growth rate despite the variation in the OOM volatility distribution among these sites (Supplementary Information Fig. 7). The enhancement in particle survival probability by the NCG-contributed initial growth is most substantial (a factor of 12 to more than 100) for polluted megacities with a high concentration of background aerosols, and it also exceeds a factor of 2 at the relatively clean boreal forest and high-altitude sites. Further consideration of the microphysical feedback associated with the coagulation sink contributed by grown new particles may buffer this enhancement yet it will not change our findings, especially for megacities. While NPF is known to be a major contributor to CCN, our results suggest that with the globally abundant condensable OOMs (Supplementary Information Fig. 8), NPF influences the regional CCN budgets more profoundly than previously thought. For example, in recent reporting of NPF in the remote upper troposphere^38^, assuming the NCG is similar to that in the planetary boundary layer, the NCG enhancement for 3 nm survival probability would be comparable to that in Hyytiälä (typically a factor of 2.5) considering the similarity in the condensation sink ( ~ 2 × 10^-3 ^s^-1^)^39^.

Besides the climate impacts, our findings address a persistent puzzle about the observed frequent NPF events in polluted urban environments. Despite highly efficient nucleation from sulfuric acid and stabilizing bases^22,37,40^, how the freshly nucleated particles can survive against the scavenging of high-concentration background aerosols was unanswered. This survival puzzle was previously explained with the co-condensation of supersaturated nitric acid and ammonia, which can dramatically boost the growth of particles in well-controlled chamber experiments^18,41^. However, even in an ideal condition that the composition, morphology, and particle-phase diffusion of new particles do not retard the uptake of condensable vapors^42,43^, nitric acid starts to dramatically boost particle growth from 3–5 nm^41^, i.e., freshly nucleated particles would have been scavenged before nitric acid can condense (Fig. 1d and Supplementary Information Fig. 5a). Our data shows that OOM condensation with a large NCG of freshly nucleated particles is plausibly a decisive mechanism to drive rapid initial growth through the most crucial sub-3 nm “death zone”, protecting them from high scavenging losses in polluted urban environments and hence increasing their plausible contributions to haze formation.

In summary, our analysis based on atmospheric measurements shows that the contribution of a large NCG (nanoparticle concentration gradient) provides an explanation for the mystery behind the rapid initial growth of freshly nucleated particles, which is critical for these particles to survive scavenging losses and subsequently influence climate and air quality. This mechanism may have even more notable implications for the future and pre-industrial climates. With lower sulfuric acid concentrations in future and pre-industrial atmospheres^44^, it is reasonable to expect increasing importance of accounting for NCG in reproducing the real new particle growth, which may correspond to an even larger fraction of contribution from NPF to regional and global CCN budgets^45^.

## Methods

### Measurements of atmospheric new particle formation

In our analysis, we evaluate the atmospheric NPF data measured at seven sites across the globe^17,21,46–49^, including Hyytiälä (61.10°N, 24.17°E, SMEAR II station, a rural site measuring NPF in Finnish boreal forest), Beijing (39.94°N, 116.30°E, BUCT station, an urban site in the Beijing-Tianjin-Hebei region), Shanghai (31.17°N, 121.43°E, SAES station, an urban site in Yangtze River Delta), Nanjing (32.12°N, 118.95°E, SORPES station, a regional background in Yangtze River Delta), Hong Kong (22.22°N, 114.25°E, CDSS station, a coastal background site in Pearl River Delta), Chacaltaya (6.35°S, 68.13°W, GAW station, a high-altitude site measuring the outlet flow of the Amazon Basin on its western edge), and Po Valley (44.65^o^ N, 11.62^o^ E, a southern European urban site in northern Italy).

NPF and air pollution have been continuously monitored at the BUCT site since 2018. Here we use the data measured from January to December 2018. During this period, we measured the number size distribution of particles ranging from 1 nm to 10 μm using a custom-made diethylene glycol scanning mobility particle spectrometer (DEG-SMPS, 1–7.5 nm)^50,51^ and a particle size distribution system (PSD, 3 nm to 10 μm)^52^. A miniature cylindrical differential mobility analyzer was deployed in the DEG-SMPS to achieve high sizing accuracy and support analysis of particle growth rate and survival probability. Sulfuric acid and OOMs were measured using nitrate CI-APi-TOFs with calibrated sensitivity and transmission efficiency^53^. The average concentration of iodic acid was significantly lower than that of sulfuric acid during the measurements^54^. Data from an iodide CI-APi-TOF and a VOCUS-PTR indicated that extra vapors that were underestimated or undetected by the nitrate CI-APi-TOF were unlikely to contribute substantially to the initial growth in Beijing. Detailed information on the BUCT site and instruments has been reported previously^21,46^.

We conducted long-term field measurements at Hyytiälä and analyzed the data from March to May 2020. During this period, a differential mobility analyzer-train (DMA-train) was deployed to measure the size distribution of sub-8 nm particles^55^. It classified particles into 6 channels based on their electrical mobility diameters, which were subsequently converted into mass diameters in our analysis^56^. Our previous studies have shown that the high sizing accuracy of the DMA-train can provide stable estimates of aerosol size distributions and benefit the evaluation of particle growth rates and survival probabilities^57^. Particles larger than 8 nm were measured using differential mobility particle spectrometers^58^. Sulfuric acid and OOMs were measured using a nitrate-ion chemical ionization atmosphere-pressure-interface time-of-flight mass spectrometer (nitrate CI-APi-TOF, Aerodyne Research Inc.)^59^. We calibrated its sensitivity using a previously reported method^60^ and the calibration uncertainty is estimated to be +100%/-50%. Detailed information on the Hyytiälä site and measurements can be found in our previous studies^17^.

A bromide CI-APi-TOF was also deployed during the measurement at Hyytiälä, but its data were available for only a limited number of days and were not included in this study. Case analysis showed that adding bromide CI-APi-TOF data increased the predicted initial growth rate, but it was still within experimental uncertainties and did not affect the conclusion of this study. Including bromide CI-APi-TOF data does not affect our findings on the importance of the NCG for sub-3 nm initial growth, and it would improve the growth rate (GR) closure for some NPF events in springtime Hyytiälä (e.g., Supplementary Information Fig. 9). A recent study^34^ also showed that for summer Hyytiälä, new particles were observed to grow slower than expected even without the NCG, suggesting unidentified growth-limiting mechanisms, particularly for the subsequent growth of particles larger than 3 nm during summertime forested areas. We note that the challenge in summertime growth rate closure does not affect the main conclusions of the study, and rather suggests some unidentified growth-limiting mechanisms in the super 3 nm size range.

We also use the sulfuric acid and OOMs measured by nitrate CI-APi-TOFs at Shanghai, Nanjing, Hong Kong, Chacaltaya, and Po Valley. The Shanghai, Nanjing, and Hong Kong datasets^47^ were collected concurrently in the autumn and winter of 2018. The Chacaltaya and Po Valley datasets^48,49^ were collected from December 2017 to May 2018 and March to April 2022, respectively.

### Determination of particle growth rate and survival probability

We evaluated the growth and survival of freshly nucleated particles using aerosol size distributions measured during strong NPF events at Hyytiälä and Beijing. These strong events were classified based on the clear pattern of particle formation and growth observed. By selecting these strong events, we minimize the uncertainties and perturbations associated with measurements, transport, and emissions. Our previous analysis has shown that transport influences sub-3 nm particles minorly at both sites, especially during strong NPF events^61,62^.

We retrieved the growth rate, formation rate, and survival probability of freshly nucleated particles using the measured aerosol size distributions. The growth rate was computed using the 50% appearance time method for its better accuracy for freshly nucleated particles than the mode-fitting method, and we have corrected for the influence of high coagulation sink on the apparent growth^63,64^. The results provide evidence for the rapid initial growth of new particles (on average ~ 3nmh-1 at Hyytiälä and Beijing). The comparison between measured and predicted particle survival probability (Fig. 3c) also supports the rapid initial growth. Previous studies have reported lower initial growth rates^23,35^, which were likely underestimated by the mode-fitting method due to the influence of continuous nucleation on the fitted mode diameter^19^. The uncertainty in the measured growth rate was characterized using the 95% confidence interval of the inverse of the fitted slope of appearance time versus particle size (Fig. 1a, b). We note that the uncertainties in the measured growth rate for a single event can be comparable to the difference between the measured growth rate and the predicted growth rate without the NCG, making it difficult to conclude a growth rate closure based on a single NPF event. However, statistical analysis of the growth rate and particle survival probability (Fig. 3) shows that the measured new particles grew faster than could be explained without the NCG.

The formation rate was retrieved using a population balance method with improved accuracy in evaluating the influence of coagulation in polluted environments^65^. The survival probability was retrieved using Eqs. 1 and 2 for Hyytiälä and Beijing, respectively.

1 $${P}_{{{\rm{meas}}}}\left({d}_{{{\rm{p}}}1}\to {d}_{{{\rm{p}}}2}\right)={n}_{\log 2}/{n}_{\log 1}$$

2 $${P}_{{{\rm{meas}}}}\left({d}_{{{\rm{p}}}1}\to {d}_{{{\rm{p}}}2}\right)={J}_{2}/{J}_{1}$$

where *P*_meas_ (-) is the measured survival probability, *d*_p_ is the particle diameter (nm), *n*_log_ is the aerosol size distribution in the logarithmic size scale (d*N*/dlog*d*_p_, cm^-3^), and *J* is the particle formation rate (cm^-3^ s^-1^). The values of *n*_log_ and *J* were obtained along the growth trajectory as indicated by the subscripts 1 and 2. Different formulae were applied for Hyytiälä and Beijing to address the difference in the evolution of aerosol populations^8^. The relative difference in the retrieved *P*_meas_ of sub-3 nm particles using Eqs. 1 and 2 is 50-100%, which does not affect our conclusion on the survival probability elevated by the rapid initial growth. Furthermore, we have recently validated the application of these equations using measured and simulated NPF events.

The theoretically predicted survival probability, *P*_theo_ (-), was computed using the growth rate and coagulation sink^66,67^ (Eq. 3).

3 $${P}_{{{\rm{theo}}}}\left({d}_{{{\rm{p}}}1}\to {d}_{{{\rm{p}}}2}\right)=\exp \left[{\int }_{{d}_{{{\rm{p}}}1}}^{{d}_{{{\rm{p}}}2}}-{{\rm{CoagS}}}\left({d}_{{{\rm{p}}}}\right)/{{\rm{GR}}}\left({d}_{{{\rm{p}}}}\right)\cdot {{{\rm{d}}}d}_{{{\rm{p}}}}\right]$$

where CoagS is the coagulation sink (s^-1^) and GR is the growth rate (nm s^-1^). The accuracy of this formula to pseudo-steady-state particle size distributions has been validated previously^8,68^.

We also reconstructed the concentration of particles larger than 3 nm using *P*_theo_ and the measured formation rate. Particles formed every 5-min period were taken as a population, and their size and concentration were theoretically predicted using the growth rate and survival probability. Summing the concentrations of different particle populations that have grown beyond 3 nm yields the reconstructed concentration. The fraction of NPF-produced CCN that would be scavenged without the contribution of NCG to sub-3 nm initial growth was determined by comparing the survival probability predicted using Eq. 3 with GR values obtained using a monodisperse model and a discrete model.

### Aerosol growth model

We modeled particle growth from a single-particle perspective based on the net condensation flux of vapors to the particle phase. Equivalent to the growth of a single particle, the simulated particles were assumed to be strictly monodisperse, i.e., every particle shares the same size and composition at the same moment. The details of monodisperse aerosol growth models have been discussed elsewhere^11,26^. The net condensation rate of vapor *i* was computed using Eq. 4,

4 $$\frac{{{\rm{d}}}{C}_{i,{{\rm{p}}}}}{{{\rm{d}}}t}={N}_{{{\rm{p}}}}{\beta }_{i,{{\rm{p}}}}\left({C}_{i,{{\rm{g}}}}-{C}_{i,{{\rm{p}}}}^{{{\rm{eq}}}}\right)={N}_{{{\rm{p}}}}{\beta }_{i,{{\rm{p}}}}\left({C}_{i,{{\rm{g}}}}-{\alpha }_{i,{{\rm{p}}}}{K}_{i,{{\rm{p}}}}{C}_{i}^{{{\rm{sat}}}}\right)$$

where the subscript *i* indicates vapor *i, C*_*i*,p_ and *C*_*i*,g_ are the mass concentrations in the particle phase and gas phase, respectively (μg m^-3^), *t* is time (s^-1^), *N*_p_ is the concentration of particles (cm^-3^), *β*_*i*,p_ is the collision coefficient between vapor *i* and the particles (cm^3^ s^-1^), *C*_*i*,p_^eq^ is the equilibrium mass concentration on the curved particle surface (μg m^-3^), *α*_*i*,p_ is the activity (-), *K*_*i*,p_ is the Kelvin term (-), and *C*_*i*_^sat^ is the saturation concentration on a flat surface of pure *i* molecules (μg m^-3^). The *α*_*i*,p_ term decreases *C*_*i*,p_^eq^ due to the solution effect characterized by Raoult’s law. The Kelvin effect described by the *K*_*i*,p_ term increases *C*_*i*,p_^eq^, where ln*K*_*i*,p_ = *d*_k_/*d*_p_ and *d*_k_ is the Kelvin diameter (nm). Particle-phase diffusion is herein assumed to be sufficiently fast, i.e., freshly nucleated particles is taken to be liquid-like such that diffusion within the particle does not significantly resist the uptake of vapors.

We accounted for sulfuric acid dimers by treating them as non-volatile vapors. However, the coagulation growth was not accounted for in the monodisperse model. This is based on the fact that the concentration of growing clusters in atmospheric conditions analyzed in this study is significantly lower than condensable vapors due to cluster evaporation and coagulation scavenging^63^. Consequently, their influence on initial growth during the observed atmospheric NPF events is negligible. For the initial growth of strongly bound clusters at low sinks, self-coagulation may contribute significantly to the initial growth^69,70^.

We used a discrete model to account for the condensational growth of a population of new particles^71^. It obtains the concentrations of particles with different sizes and compositions by solving a series of population balance equations. To be consistent with the monodisperse model, coagulation between nanoparticles is not accounted for, and sulfuric acid dimers are treated as non-volatile vapors. The net flux of the mass concentration from size p to p + 1 due to vapor *i* condensation (μg m^-3^ s^-1^) can be expressed as

5a $$\frac{{{\rm{d}}}{C}_{i,{{\rm{p}}}}}{{{\rm{d}}}t}={N}_{{{\rm{p}}}}{\beta }_{i,{{\rm{p}}}}{C}_{i,{{\rm{g}}}}-{N}_{{{{\rm{p}}}}_{+1}}{\beta }_{i,{{{\rm{p}}}}_{+1}}{C}_{i,{{{\rm{p}}}}_{+1}}^{{{\rm{eq}}}}$$

5b $$\frac{{{\rm{d}}}{C}_{i,{{\rm{p}}}}}{{{\rm{d}}}t}=\underbrace{{N}_{{{\rm{p}}}}{\beta }_{i,{{\rm{p}}}}\left({C}_{i,{{\rm{g}}}}-{C}_{i,{{\rm{p}}}}^{{{\rm{eq}}}}\right)}_{{{\rm{single}}}\; {{\rm{particle}}}\; {{\rm{term}}}}+\underbrace{{N}_{{{\rm{p}}}}{\beta }_{i,{{\rm{p}}}}\left({C}_{i,{{\rm{p}}}}^{{{\rm{eq}}}}-\frac{{N}_{{{{\rm{p}}}}_{+1}}{\beta }_{i,{{{\rm{p}}}}_{+1}}}{{N}_{{{\rm{p}}}}{\beta }_{i,{{\rm{p}}}}}{C}_{i,{{{\rm{p}}}}_{+1}}^{{{\rm{eq}}}}\right)}_{{{\rm{NCG}}}\; {{\rm{term}}}}$$

where the subscript p_+1_ indicates particles containing one more *i* molecule than particles p, and the minus sign before d*C*_*i*,p_/d*t* indicates that condensational growth causes the decrease of *C*_*i*,p_. Equation 5a describes the fact that the growth flux of particle p to a p_+1_ by the vapor *i* condensation is determined by its association with *i* and the dissociation from p_+1_. Equation 5b is derived by rearranging Eq. 5a, and it emphasizes that the NCG term can accelerate the growth of a population of particles compared to a single particle. The magnitude of the NCG is affected by the size-dependent dissociation rate and the coagulation sink, which in turn affects the NCG term in Eq. 5b. Other equivalent expressions can be found in previous studies^27,28,33^.

The saturation concentration of measured OOMs was estimated from the number of constituent carbon (nC), oxygen (nO), nitrogen (nN), and hydrogen (nH) atoms in a molecule using the volatility basis set (VBS) parameterization^72^. Sulfuric acid was considered non-volatile. Vapors with saturation concentrations were grouped before being used as the inputs for the monodisperse and discrete models. The parameterization varies with the governing functional group of the detected OOMs. Therefore, we use different parameterizations for different sites to account for differences in OOM source and oxidation processes. The parameterization in Li et al.^73^ was used for Hyytiälä, Chacaltaya, and Po Valley. A workflow that applies parameterization according to the potential precursors of OOMs^47^ was used for Beijing, Shanghai, Nanjing, and Hong Kong. The influence of temperature on the volatility was corrected. The uncertainty associated with the parameterization was estimated to be from -23% to +65% in terms of particle growth rate in Beijing. By shifting the volatility distribution by one order of magnitude, we show that the contribution of the large NCG term to the initial growth is not sensitive to the uncertainties associated with the VBS parameterization (Supplementary Information Fig. 10). That is, the uncertainties from VBS parametrization, and similarly, other sources, are unlikely to affect our main findings on the rapid initial growth enhanced by a large concentration gradient.

The initial growth rate was predicted using the monodisperse model and the discrete model. Condensable OOMs were classified into bins based on volatility. The monodisperse model tracked the composition, size, and concentration of a strictly monodisperse aerosol population, and then derived the growth rate using the predicted size as a function of time. The discrete model also takes non-volatile species (namely, sulfuric acid) and volatility-classified OOMs as the input condensing vapors. To address the high computational expense of the discrete model, we assumed that particles with the same diameter had the same compositions and the mass difference between two adjacent bins is taken as the average mass of OOMs. The model was initialized with a pure sulfuric acid particle, and then numerically solved for the size-resolved particle concentration in Eq. 5 for pseudo-steady-state values. The growth rate was then determined using the growth flux. This is practically achieved by 1) initialize the condensation vapors with fixed concentrations; 2) initialize the particle size distribution with zero NCG, i.e., assuming that the same number concentration for every discrete bin; 3) update the mole fraction of vapor molecules in every bin; 4) compute the growth rate contributed by condensing vapor molecules; 5) update the particle size distribution using growth rate and coagulation sink; 6) repeat steps 3-5 until convergence. We have validated the discrete model using an idealized system containing two condensable vapors, for which the instantaneous cluster concentration can be solved without these simplifications.

### Growth rate of an aerosol population

The net flux of growing particles by the condensation of vapor *i* can be equivalently expressed in terms of the growth rate of a collection of particles, as given in Eq. 6^27,28^

6 $${{\rm{GR}}}=\underbrace{\Delta{d}_{{{{\rm{p}}}}_{+1}}\left({\beta }_{i,p}{N}_{i}-{\gamma }_{p}\right)}_{{{\rm{single}}}\; {{\rm{particle}}}\; {{\rm{term}}}}+\underbrace{\Delta{d}_{{{{\rm{p}}}}_{+1}}\frac{1}{{N}_{p}}\left({\gamma }_{p}{N}_{{{\rm{p}}}}-{\gamma }_{{{{\rm{p}}}}_{+1}}{N}_{{{{\rm{p}}}}_{+1}}\right)}_{{{\rm{NCG}}}\; {{\rm{term}}}}$$

where *d*_*i*_ and *d*_p_ are the diameters of vapor *i* and particles, respectively, ∆*d*_p+1_ is the size increasing of particle p upon the uptake of an i molecule, *β*_*i*,p_ is the collision coefficient between *i* and particles (cm^3^ s^-1^), and *γ*_p+1_ is the dissociation rate of *i* from particles containing one more *i* molecule than particles p (s^-1^). Equation 6 can be derived from Eq. 5 by expressing the equilibrium concentration in the form of dissociation rate and replacing the growth flux expressed in terms of vapor mass by the size increase ∆*d*_p+1_. The value of *γ* was obtained using the following equation,

7 $${\gamma }_{{{{\rm{p}}}}_{+1}}={\beta }_{i,p}{N}^{{{\rm{\theta }}}}\exp \left(\frac{{\Delta }_{{{\rm{r}}}}{G}_{{{\rm{m}}}}^{{{\rm{\theta }}}}}{{RT}}\right)$$

where Δ_r_*G*_m_^θ^ is the standard partial molar reaction Gibbs free energy of the condensation process, i.e., binding energy (J mol^-1^), *N*^θ^ is the standard concentration (cm^-3^) defined at the standard pressure, *R* is ideal gas constant (J K^-1^ mol^-1^), and *T* is temperature (K). The value of Δ_r_*G*_m_^θ^ is assumed to follow the Kelvin equation and the Raoult’s law for ideal liquids, as accurate higher-level thermochemistry methods^74^ are currently not available for the condensation of organic molecules.

### Earth system model

The data used in this study, namely the condensation sink and the LVOC concentration, are derived from the Norwegian Earth System Model version 2 (NorESM2)^75^. Note that we did not predict CCN concentrations using NorESM2 in this study. Simulations were executed for the years 2015–2018, using the emission scenario SSP2-4.5. Nudging of surface pressure and horizontal winds was applied to ERA-Interim reanalysis data^76^ with a relaxation time of 6 hours. The simulations employed sea surface temperature and sea ice data from the Hadley Centre Sea Ice and Sea Surface Temperature dataset^77^. The details of simulations can be found in Blichner et al.^78^.

The land model employed in NorESM2 is the Community Land Model version 5 (CLM5)^79^ run in bio-geochemistry configuration with prognostic crop modeling. Emissions of biogenic volatile organic compounds in CLM5 are calculated using the Model of Emissions of Gases and Aerosols from Nature version 2.1^80^, which is integrated within CLM5. Secondary organic aerosol in the model is formed through the oxidation of monoterpene and isoprene with a molar yield of 5%, 15%, and 5%, respectively, for reactions with OH radicals, NO_3_ and O_3_. Only reactions between O_3_ and monoterpene yield low volatility vapors in the model^81^.

## Supplementary information

Supplementary Information

Transparent Peer Review file

## Data Availability

The full dataset shown in the figures in the main text and supplementary materials is publicly available at 10.5281/zenodo.13761850.81

## Code availability

The monodisperse aerosol growth model for growth rate from a volatility basis set of oxygenated organic molecules is publicly available at 10.5281/zenodo.13761850.81
