# Supplementary material for: The key role of nanoparticle concentration gradient in aerosol initial growth
Source: Nat Commun. 2026 Mar 2;17:3338. doi: 10.1038/s41467-026-70082-2 (PMC13065832; doi:10.1038/s41467-026-70082-2)
Supplement: Supplementary file 1 — Supplementary Information [file 41467_2026_70082_MOESM1_ESM.pdf]

Supplementary Information for

The key role of nanoparticle concentration gradient in aerosol initial growth

Runlong Cai[1,2,3*], Xiaoxiao Li[4], Yuyang Li[5], Sara Blichner[6,7], Dominik Stolzenburg[2,8], Qiaozhi Zha[2,9], Jing Cai[2,10], Wei Nie[9], Chao Yan[9], Dan Dan Huang[11], Zhe Wang[12], Jin Wu[5], Rujing Yin[13], Nina Sarnela[2], Wei Huang[2,14], Santeri Tuovinen[2], Sebastian Holm[2], Lauri Ahonen[2], Lei Yao[1], Aijun Ding[9], Federico Bianchi[2], Yongchun Liu[15], Paul Winkler[16], Tuukka Petäjä[2], Jianmin Chen[1], Veli-Matti Kerminen[2], Lin Wang[1], Douglas Worsnop[2,17], Jingkun Jiang[5*], Markku Kulmala[2,9,15*], Juha Kangasluoma[2]

[1]Shanghai Key Laboratory of Atmospheric Particle Pollution and Prevention (LAP[3]), Department of Environmental Science & Engineering, Fudan University, Shanghai, 200438, China

[2]Institute for Atmospheric and Earth System Research / Physics, Faculty of Science, University of Helsinki, Helsinki, 00014, Finland

[3]IRDR ICoE on Risk Interconnectivity and Governance on Weather/Climate Extremes Impact and Public Health, Fudan University, Shanghai, 200438, China

[4]School of Resource and Environmental Sciences, Wuhan University, Wuhan, 430072, China

[5]State Key Laboratory of Regional Environment and Sustainability, School of Environment, Tsinghua University, 100084 Beijing, China

[6]Bolin Centre for Climate Research, Stockholm University, SE-106 91 Stockholm, Sweden

[7]Department of Environmental Science, Stockholm University, Stockholm SE-106 91, Sweden

[8]Institute of Materials Chemistry, TU Wien, Vienna, 1060, Austria

[9]Joint International Research Laboratory of Atmospheric and Earth System Research, School of Atmospheric Sciences, Nanjing University, Nanjing, 210023, China

[10]School of Atmospheric Physics, Nanjing University of Information Science and Technology, Nanjing, 210044, China

[11]State Environmental Protection Key Laboratory of Formation and Prevention of Urban Air Pollution Complex, Shanghai Academy of Environmental Sciences, Shanghai, 200003, China

[12]Division of Environment and Sustainability, The Hong Kong University of Science and Technology, Hong Kong, 999077, China

[13]Key Laboratory of Industrial Ecology and Environmental Engineering (Ministry of Education), School of Environmental Science and Technology, Dalian University of Technology, Dalian, 116024, China

[14]Now at: PSI Center for Energy and Environmental Sciences, 5232 Villigen PSI, Switzerland

[15]Aerosol and Haze Laboratory, Beijing Advanced Innovation Center for Soft Matter Science and Engineering, Beijing University of Chemical Technology, Beijing, 100029, China

[16]Faculty of Physics, University of Vienna, Vienna, 1090, Austria

[17]Aerodyne Research Inc., 45 Manning Road, Billerica, Massachusetts, 01821, USA

*Corresponding author. Email: runlong_cai@fudan.edu.cn (R.C.); jiangjk@tsinghua.edu.cn (J.J.); markku.kulmala@helsinki.fi (M.K.)

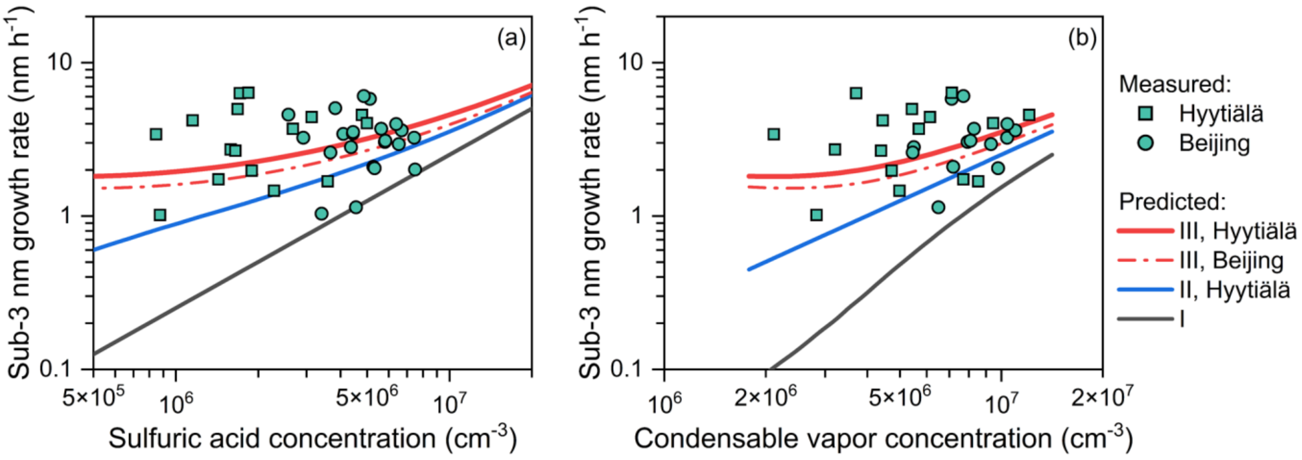

Supplementary Information Fig. 1 | The mystery of initial growth.

The horizontal axis shows the measured concentration of (a) sulfuric acid and (b) extremely low-volatile vapors (sulfuric acid and OOMs with extremely low volatility). The uncertainty in the measured growth rate was estimated to be 50%-100%. I, II, and III represent the predicted growth by sulfuric acid, growth by sulfuric acid and OOMs without NCG, and growth by sulfuric acid and OOMs with the contribution of the NCG term, respectively. The lines show the predicted growth rate using condensable vapors. The input concentration of low-volatile OOMs for scenario III was determined according to its correlation with the sulfuric acid concentration. As only a weak correlation was observed between OOMs and sulfuric acid in both Hyytiälä and Beijing, we used the average concentration of low-volatile OOMs during the campaign as a constant input value. This figure shows that the observed high initial growth rate and its weak dependence on condensable vapor concentration cannot be explained by previous understandings, while it can be addressed using the overlooked NCG.

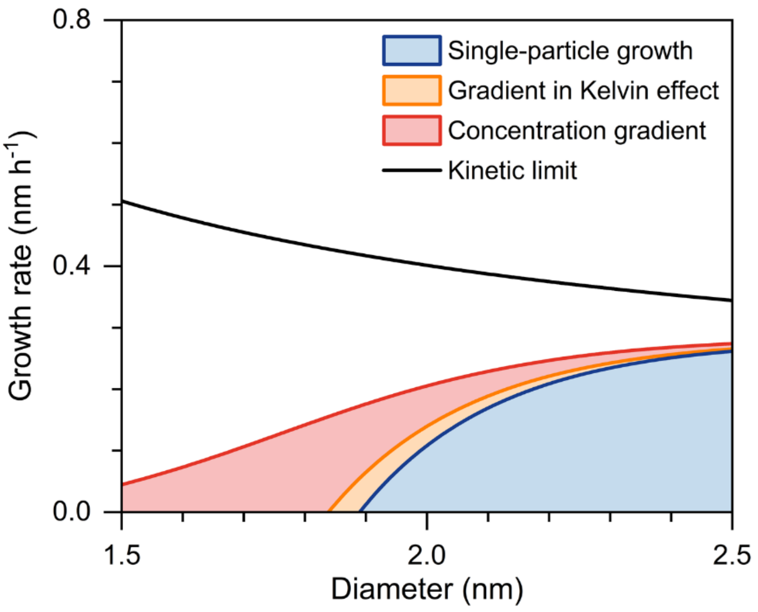

Supplementary Information Fig. 2 | Contribution of different mechanisms to the initial growth of new particles.

The result was obtained by simulating the growth of stable sulfuric acid clusters by the condensation of a volatile vapor. The molecular mass, density, concentration, and saturation concentration of the volatile vapor were assumed to be 300 g mol^{-1}, 1400 kg m^{-3}, 10^{6} cm^{-3}, and 2×10^{3} cm^{-3} (C^{*} = 10^{-6} μg m^{-3}), respectively. The single particle growth is determined by the competition between the vapor association and dissociation with respect to particles of the same size and composition. The gradient of the Kelvin effect ($\gamma_n N_n - \gamma_{n+1} N_n$, see Eq. 10) accounts for the smaller dissociation rate from larger particles. The NCG term ($\gamma_{n+1} N_n - \gamma_{n+1} N_{n+1}$) accounts for the lower concentration of larger particles and correspondingly smaller dissociation flux. The growth at the kinetic limit assumes no dissociation of vapors from particles.

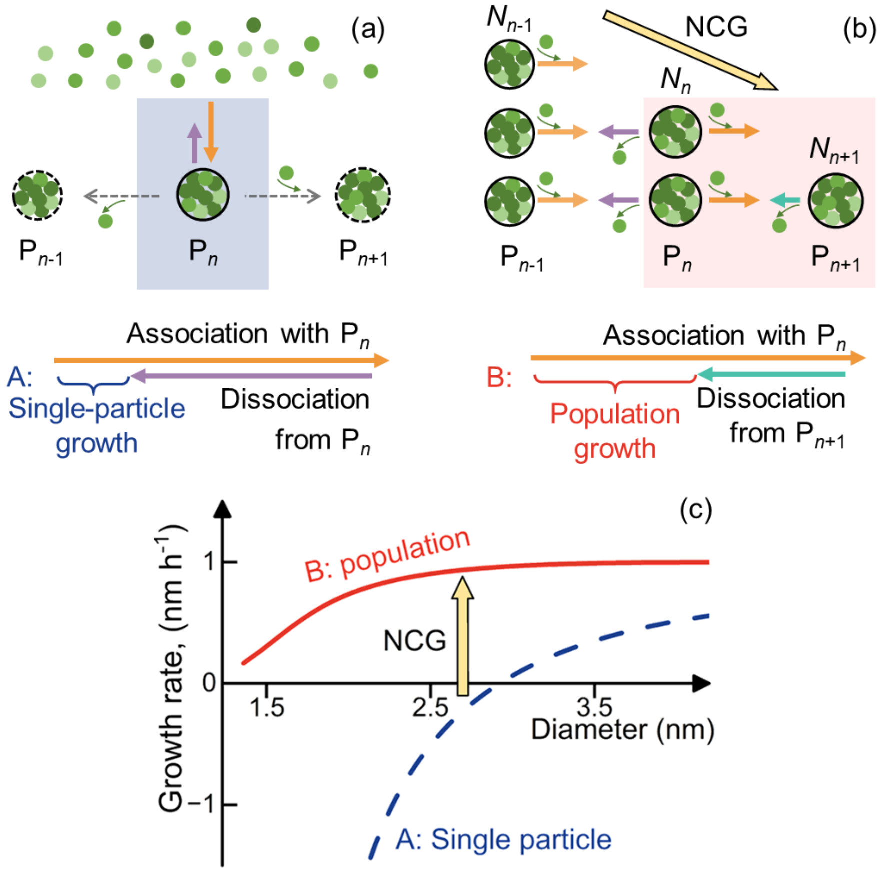

Supplementary Information Fig. 3 | A kinetic view of the growth of a single particle and a population of particles.

The yellow arrow indicates the NCG term and its influences on the initial growth. (a) schematics illustrating the growth flux in a single-particle perspective. P_n represents particles containing n molecules. Green circles represent different condensable molecules. The solid arrows indicate association and dissociation processes. The shaded area indicates the balance that determines the growth of a single particle, i.e., the mass balance is evaluated for a single particle, and it is determined by the condensation flux onto this particle (orange arrow) and the evaporation flux from this particle (purple arrow). (b) schematics illustrating the growth flux of a population of particles. N_n is the concentration of particles P_n. The shaded area indicates the balance that determines the growth of a population of nanoparticles, i.e., the mass balance is evaluated for particles with different sizes, and it is determined by the condensation flux onto particle P_n (orange arrow) and the evaporation flux from P_{n+1} (purple arrow). (c) schematics illustrating the growth rate of a growing aerosol population.

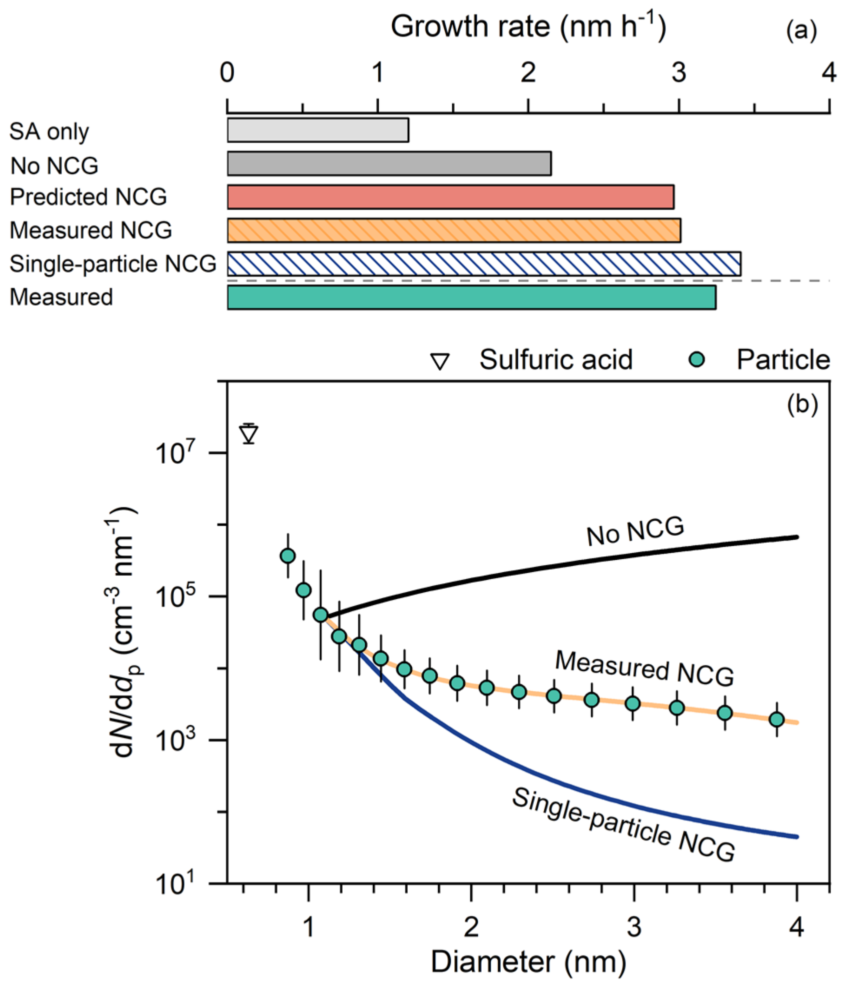

Supplementary Information Fig. 4 | Average initial growth rate of sub-3 nm particles in Beijing.

(a) Measured and predicted growth rates. The horizontal dashed line is used to separate the measured and predicted growth rates. (b) Particle size distribution corresponding to different NCG. We also convert the concentration of sulfuric acid to $\mathrm{d}N/\mathrm{d}d_\mathrm{p}$ using a previously reported method [81]. The horizontal axis is the geometric diameter.

This figure shows that in addition to the consistency in the measured and predicted growth rate in Fig. 3, the measured particle size distribution indicates a higher growth rate that coincides with the "missing" growth rate. This provides further support for our finding that the overlooked contribution of the NCG term explains the gap between previous understanding and atmospheric observations.

In this figure, "SA only" indicates the growth rate predicted by sulfuric acid condensation. "Measured" indicates the growth rate retrieved from particle size distributions. "No NCG" indicates single-particle growth by OOMs and sulfuric acid without the contribution of NCG. This is equivalent to explicitly assuming a constant particle number concentration as a function of the number of molecules per

particle. Correspondingly, the dN/dd_p in (b) increases with particle size.

"Predicted NCG" is the NCG predicted from vapor concentrations and scavenging losses. The size distribution is obtained by iteratively solving the discrete model for population growth (indicated by Eq. 5), with an initial guess that the NCG is equal to the "single-particle NCG". In every iteration, the growth rate is computed using the NCG, and then the NCG is updated such that the growth, evaporation, and scavenging losses of particles reach a pseudo-steady-state. As a lower NCG would lead to a smaller growth rate, which in turn decreases the survival probability and hence increases the NCG, the predicted NCG converges quickly after a few iterations.

"Measured NCG" is derived from the measured particle size distribution. Average size distribution is used herein to reduce the influence of uncertainties in sub-3 nm particle measurements on growth rate prediction. The growth rate is then predicted using this measured distribution.

"Single-particle NCG" is obtained using the predicted survival probability with the single-particle growth rate, i.e., the survival probability is first derived using the predicted single-particle growth rate and loss rate, and then NCG is determined using the survival probability. The single-particle NCG is expected to overestimate the NCG due to an underestimation of the initial growth rate.

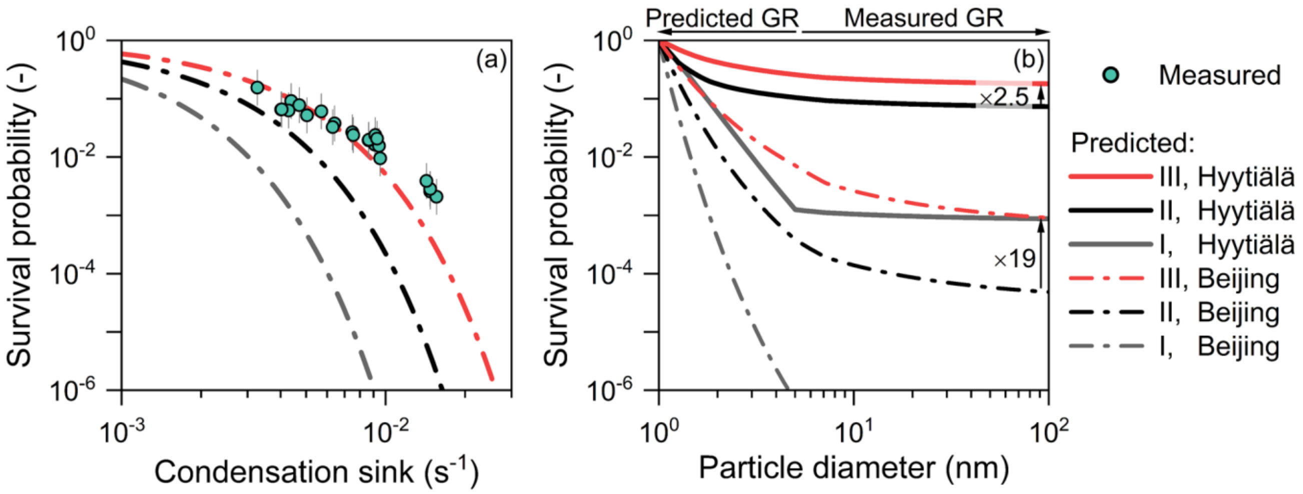

Supplementary Information Fig. 5 | Survival probability of new particles in Beijing and Hyytiälä. (a), the measured and theoretically predicted survival probabilities of sub-3 nm new particles in Beijing. The uncertainty bar indicates a +100% / -50% uncertainty in the measured survival probability. The theoretical survival probabilities are predicted using the mean values of predicted growth rates in Fig. 3b. I, II, and III represent growth by sulfuric acid, growth by sulfuric acid and OOMs without NCG, and growth by sulfuric acid and OOMs with NCG, respectively. (b), The theoretically predicted survival probabilities of new particles to different sizes at the measured mean condensation sink. The I, II, and III correspond to the predicted growth of sub-5 nm particles by different mechanisms. The same measured growth rates are used for particles larger than 5 nm. The arrows indicate the enhancement by the NCG on the survival probability of particles to 100 nm.

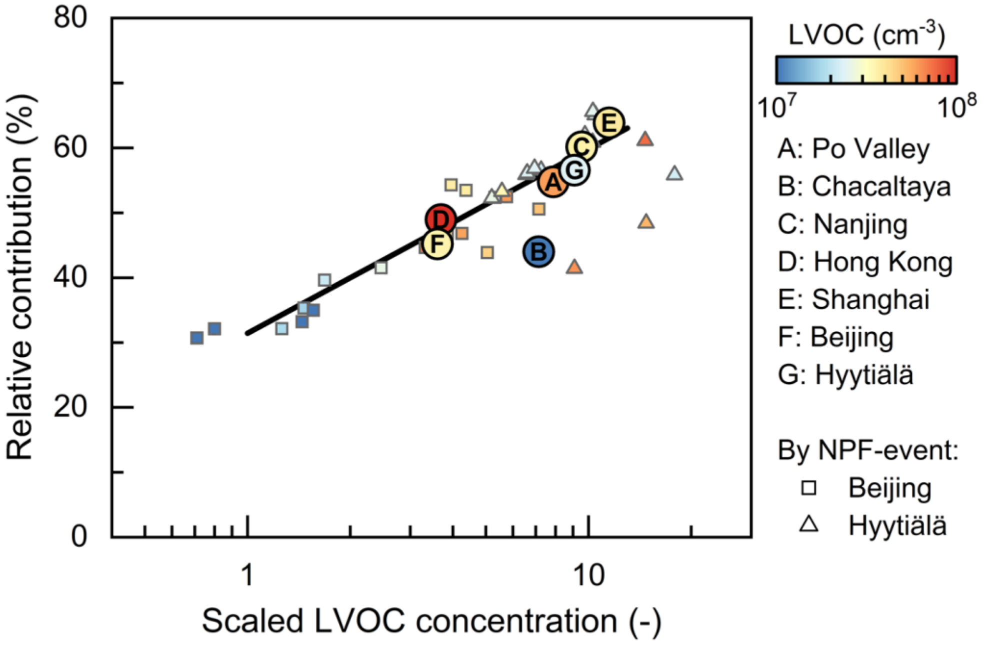

Supplementary Information Fig. 6 | Relative contribution of the nanoparticle concentration gradient to the initial growth rate.

The vertical axis indicates the ratio of the growth rate contributed by the NCG to the total condensational growth rate of sub-3 nm particles. The horizontal axis indicates the ratio of LVOC concentration to the total concentration of vapors with negligible evaporation for particle growth, including sulfuric acid and ultra-low-volatility organic compounds (saturation mass concentration C^* $< 10^{-8.5}$ μg m^{-3}). The large markers indicate the average contribution of the NCG at different sites. This contribution of the NCG to the initial growth rate as a function of the scaled LVOC concentration could serve as an estimation for the implementation in Earth system models.

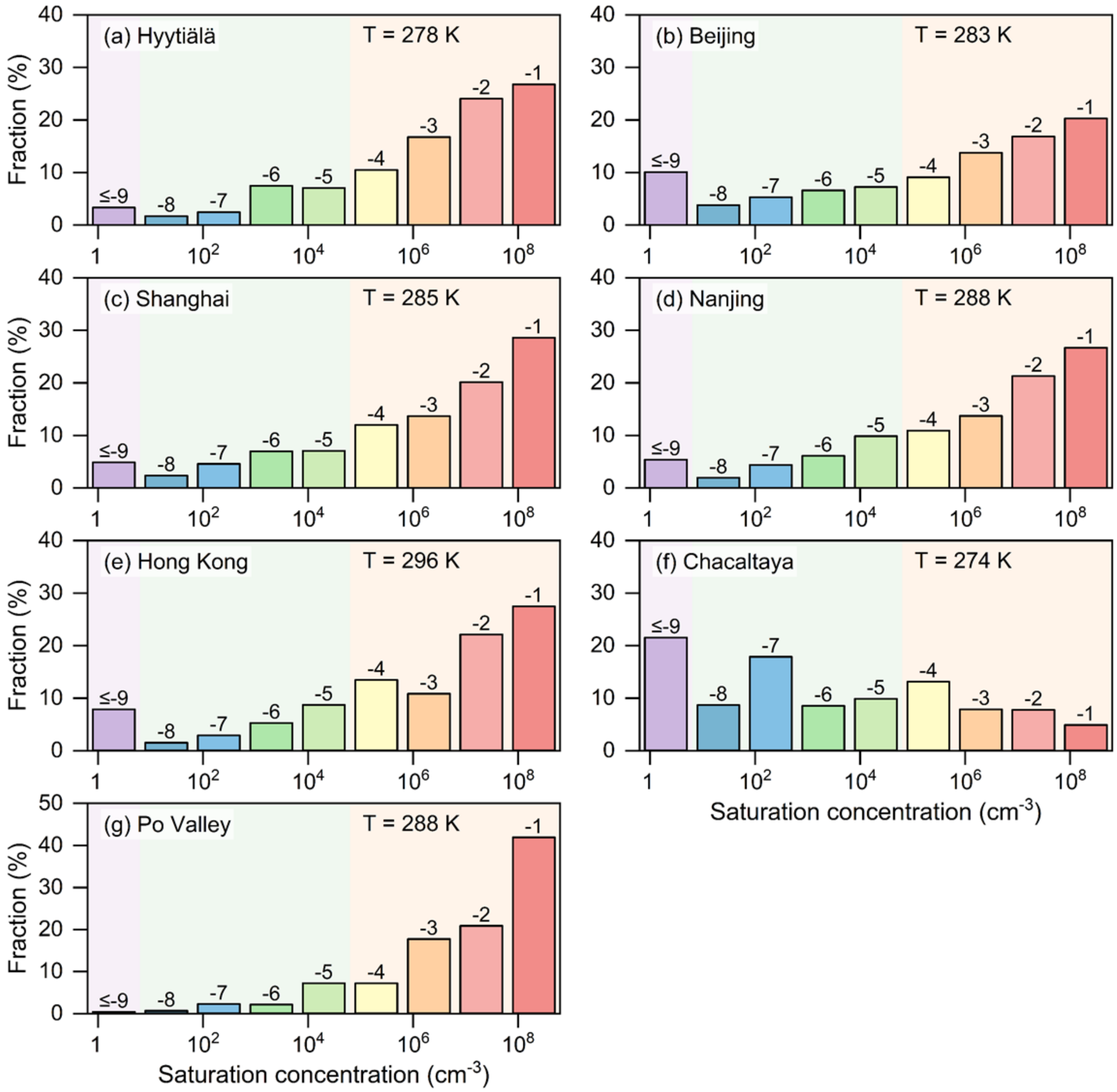

Supplementary Information Fig. 7 | Measured volatility distribution of OOMs from different sites.

(a) Hyytiälä; (b) Beijing; (c) Shanghai; (d) Nanjing; (e) Hong Kong; (f) Chacaltaya; (g) Po Valley. The bar labels indicate the saturation mass concentration (C^* μg m^{-3}) in log$_{10}C^*$. The influence of temperature on C^* has been taken into account. The shaded background indicates VBS volatility classes.

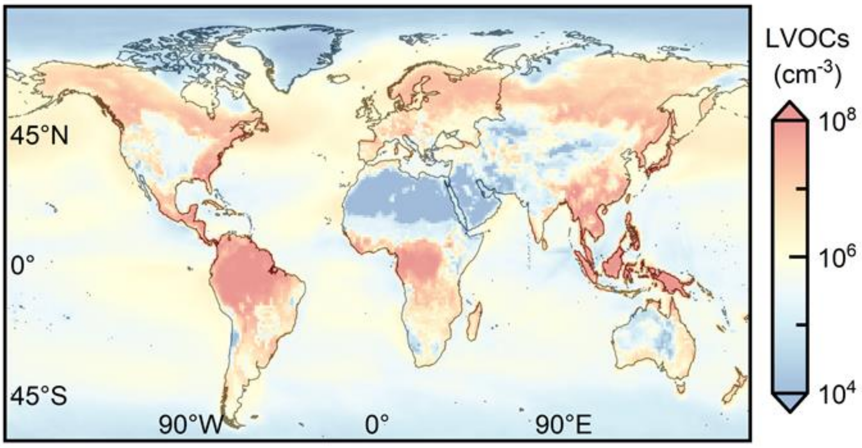

Supplementary Information Fig. 8 | Modelled spatial distribution of LVOCs.

The LVOC herein is shown in the annual average daily maximum concentration. It includes organic compounds with C^* ranging from $10^{-4.5}$ to $10^{-0.5}$ μg m^{-3} and those with lower C^* values. According to Supplementary Information Fig. 6, the LVOC concentration is herein used as an indicator for condensable OOMs.

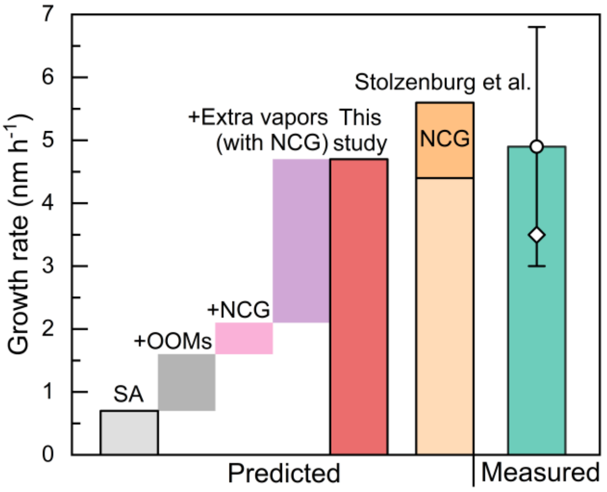

Supplementary Information Fig. 9 | A case study on the potential contribution from extra vapors to initial growth rate at the Hyytiälä site.

The NPF event was measured on 11th April 2020. Extra vapors indicate species measured by a bromide CI-APi-TOF. The floating bars indicate the contribution from different mechanisms to the initial growth. This study and Stolzenburg et al.[34] used slightly different Kelvin parameters for model predictions, and the sub-3 nm initial growth rate retrieved from measured particle size distributions (indicated by diamond and circle markers, respectively) are different yet agree with each within the uncertainty range. The standard deviation of the measured growth rate herein was obtained by comparing the results from different instruments and the growth rate retrieval methods, as described in Stolzenburg et al.[34] This figure indicates that for some NPF events, extra vapors that are undetected by nitrate CI-APi-TOF are also expected to contribute to the initial growth, and may bring the predicted growth rate closer to measured values. The relative importance of this contribution varies with events; however, similar to the discussion in Supplementary Information Fig. 10 below, it does not affect the importance of the NCG to the initial growth.

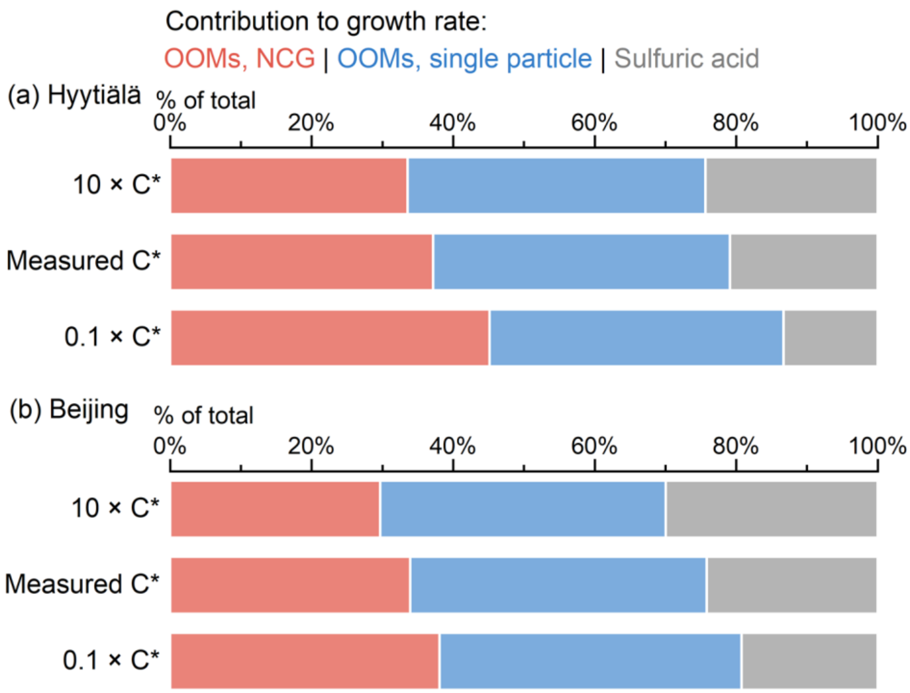

Supplementary Information Fig. 10 | Sensitivity of the growth by the large nanoparticle concentration gradient to the uncertainties in volatility basis set parameterizations.

175 The horizontal bars indicate the relative contributions of the NCG term, OOM condensation onto a single particle, and sulfuric acid condensation at (a) Hyytiälä and (b) Beijing. The measured C^* indicates the volatilities obtained with the VBS parameterizations. We applied a one-order-of-magnitude uncertainty to the volatility assignment ($10\times C^*$ and $0.1\times C^*$) to test the sensitivity of aerosol growth simulations. The result shows that the importance of the NCG of freshly nucleated particles to
180 their growth is robust against uncertainties.

Supplementary Information Table 1 | New particle formation days in Hyytiälä and Beijing.

CS is the condensation sink of sulfuric acid. GR_{SA} is the initial growth rate contributed by sulfuric acid. $GR_{SA+OOMs}$ accounts for SA and OOM condensation.

	Date (y-m-d)	Initial GR (nm h^{-1})	CS ($\times10^{-3}$ s^{-1})	SA ($\times10^{6}$ cm^{-3})	GR_{SA} (nm h^{-1})	$GR_{SA+OOMs}$ (nm h^{-1}) without NCG	with NCG
Hyytiälä	2020-03-07	4.1	2.1	0.5	0.1	0.6	1.5
	2020-03-20	3.0	0.8	1.9	0.5	1.0	1.3
	2020-03-22	2.0	1.6	4.1	1.0	2.1	2.7
	2020-04-01	2.6	0.5	1.3	0.3	1.0	1.6
	2020-04-05	3.4	0.5	2.3	0.5	1.4	1.9
	2020-04-09	1.4	0.5	1.6	0.4	1.2	1.9
	2020-04-11	3.5	0.9	2.8	0.7	1.6	2.1
	2020-04-16	2.3	1.5	1.1	0.3	0.8	1.3
	2020-04-18	1.5	0.8	2.7	0.6	1.6	2.2
	2020-04-21	5.5	1.0	2.6	0.6	3.4	6.9
	2020-04-23	3.6	1.2	1.8	0.4	2.3	4.5
	2020-04-25	3.4	1.3	1.6	0.4	1.2	2.0
	2020-05-02	5.9	5.9	4.8	1.1	3.9	6.1
	2020-05-07	6.2	1.6	5.0	1.2	2.7	3.5
Beijing	2018-01-23	1.0	4.0	3.4	0.8	-	-
	2018-01-24	1.1	9.1	4.6	1.1	1.3	1.8
	2018-01-25	3.2	14.8	7.4	1.8	2.1	2.7
	2018-01-30	3.7	9.5	-	-	-	-
	2018-01-31	2.9	9.4	6.5	1.6	2.2	3.5
	2018-02-01	-	33.6	7.9	1.9	2.7	4.3
	2018-02-02	3.0	7.5	5.8	1.4	1.6	2.0
	2018-02-03	-	7.5	5.7	1.4	1.5	2.0

2018-02-04	-	38.3	11.6	2.8	3.2	4.3
2018-02-05	5.8	8.6	5.1	1.2	1.5	2.1
2018-02-06	-	52.8	8.0	1.9	2.5	6.9
2018-02-07	3.1	8.6	5.8	1.4	-	-
2018-02-09	6.1	6.4	4.9	1.2	-	-
2018-02-10	2.1	6.3	5.3	1.3	-	-
2018-02-11	2.8	5.0	4.4	1.0	-	-
2018-02-12	2.6	4.4	3.7	0.9	-	-
2018-02-16	3.6	9.1	6.7	1.6	-	-
2018-02-20	4.0	9.3	6.4	1.5	-	-
2018-02-24	3.7	14.8	5.6	1.4	-	-
2018-03-01	2.1	5.7	5.3	1.3	-	-
2018-03-08	-	10.9	2.9	0.7	2.6	5.4
2018-10-27	3.4	14.3	4.1	1.0	2.3	3.2
2018-10-31	-	14.0	2.3	0.6	1.2	3.2
2018-11-21	3.2	4.7	2.9	0.7	-	-
2018-11-27	4.6	3.3	2.6	0.6	-	-
2018-12-06	4.1	4.3	-	-	-	-
2018-12-07	1.9	4.0	-	-	-	-
2018-12-22	-	14.3	4.5	1.1	1.4	2.2
2019-01-19	2.0	7.5	-	-	-	-
2019-01-20	3.5	3.6	4.4	1.1	-	-
2019-01-31	3.5	5.7	4.4	1.0	-	-
2019-03-06	5.1	5.0	3.8	0.9	-	-
2019-03-12	2.0	4.4	7.5	1.8	-	-
