# Supplementary material for: The key role of nanoparticle concentration gradient in aerosol initial growth
Source: Nat Commun. 2026 Mar 2;17:3338. doi: 10.1038/s41467-026-70082-2 (PMC13065832; doi:10.1038/s41467-026-70082-2)
Supplement: Supplementary file 2 — Transparent Peer Review file [file 41467_2026_70082_MOESM2_ESM.pdf]

nature portfolio

Peer Review File

The key role of nanoparticle concentration gradient in aerosol initial growth

Corresponding Author: Dr Runlong Cai

This manuscript has been previously reviewed at another journal. This document only contains information relating to versions considered at Nature Communications.

This file contains all reviewer reports in order by version, followed by all author rebuttals in order by version.

Version 1:

Reviewer comments:

Reviewer #1

(Remarks to the Author)
The authors have provided comprehensive responses to the comments from me.

Reviewer #2

(Remarks to the Author)
-

Open Access This Peer Review File is licensed under a Creative Commons Attribution 4.0 International License, which permits use, sharing, adaptation, distribution and reproduction in any medium or format, as long as you give appropriate credit to the original author(s) and the source, provide a link to the Creative Commons license, and indicate if changes were made.

In cases where reviewers are anonymous, credit should be given to 'Anonymous Referee' and the source.
The images or other third party material in this Peer Review File are included in the article’s Creative Commons license, unless indicated otherwise in a credit line to the material. If material is not included in the article’s Creative Commons license and your intended use is not permitted by statutory regulation or exceeds the permitted use, you will need to obtain permission directly from the copyright holder.
To view a copy of this license, visit https://creativecommons.org/licenses/by/4.0/

Responses to Reviewers' Comments on *Nature Communications* Manuscript NCOMMS-25-98492-A

(The key role of nanoparticle concentration gradient in aerosol initial growth)

We have revised the manuscript according to the editorial comments in the Author Checklist. Specifically, we added explanations to the method sections to further clarify the potential limitations and uncertainties in the measurements and explain how the model was validated. There are no remaining comments from reviewers.

Reviewer #1

The authors have provided comprehensive responses to the comments from me.

Response: We thank the reviewer again for providing constructive comments that have improved the manuscript.

Reviewer #2

[No remarks for authors]
